# Concealed Face Analysis and Facial Reconstruction via a Multi-Task Approach and Cross-Modal Distillation in Terahertz Imaging

**DOI:** 10.3390/s26041341

**Published:** 2026-02-19

**Authors:** Noam Bergman, Ihsan Ozan Yildirim, Asaf Behzat Sahin, Hakan Altan, Yitzhak Yitzhaky

**Affiliations:** 1 Department of Electro-Optical Engineering, School of Electrical and Computer Engineering, Ben-Gurion University of the Negev, 1 Ben-Gurion Blvd, Beer Sheva 8410501, Israel; noamberg@post.bgu.ac.il; 2 SDT Space and Defence Tech. Inc., SATGEB-2 Titanyum C Blok İhsan Dogramaci Bulv No 37, 06800 Ankara, Turkey; 3 Department of Electrical Electronics Engineering, Ankara Yıldırım Beyazıt University, 06010 Ankara, Turkey; 4 Department of Physics, Middle East Technical University, 06800 Ankara, Turkey

**Keywords:** terahertz imaging, facial biometrics, multi-task learning, knowledge distillation, cross-modal fusion, deep learning, THz facial reconstruction

## Abstract

Terahertz (THz) sub-millimeter wave imaging offers unique capabilities for stand-off biometrics through concealment, yet it suffers from severe sparsity, low resolution, and high noise. To address these limitations, we introduce a novel unified Multi-Task Learning (MTL) network centered on a custom shared U-Net-like THz data encoder. This network is designed to simultaneously solve three distinct critical tasks on concealed THz facial data, given a limited dataset of approximately 1400 THz facial images of 20 different identities. The tasks include concealed face verification, facial posture classification, and a generative reconstruction of unconcealed faces from concealed ones. While providing highly successful MTL results as a standalone solution on the very challenging dataset, we further studied the expansion of this architecture via a cross-modal teacher-student approach. During training, a privileged visible-spectrum teacher fuses limited visible features with THz data to guide the THz-only student. This distillation process yields a student network that relies solely on THz inputs at inference. The cross-modal trained student achieves better latent space in terms of inter-class separability compared to the single-modality baseline, but with reduced intra-class compactness, while maintaining a similar success in the task performances. Both THz-only and distilled models preserve high unconcealed face generative fidelity.

## 1. Introduction

Imaging in the sub-millimeter wave (Sub-MMW) or Terahertz (THz) frequency ranges has emerged as a powerful technology for security and surveillance applications. A key benefit of THz radiation is its non-ionizing nature, making it safe for screening people [1]. The characteristics of THz radiation make it usable in various applications in medical imaging [2] and industrial quality control [3]. Its primary advantage in security applications lies in the ability of THz waves to penetrate common clothing and other non-metallic, non-polar dielectric materials, revealing objects or features concealed underneath [4]. THz imaging has been demonstrated for biometric applications like facial recognition, even under occluding materials like masks, scarves, or balaclavas.

Prior research has confirmed that active imaging, particularly in the 340 GHz frequency band, can successfully penetrate common concealing fabrics to capture person-specific facial structures, thereby motivating the development of advanced biometric verification systems [5]. Early stage approaches in this domain often relied on traditional computer vision techniques, such as template matching [6,7]. These methods, sometimes using metrics like the Structural Similarity Index (SSIM), operate by performing a pixel-wise or structural comparison of a concealed face image against a gallery of pre-recorded, unconcealed reference images. While this established a baseline capability, the performance of template matching is fundamentally limited [8]. These methods are highly sensitive to variations in a subject’s posture, a challenge typically addressed in the visible domain by geometric frontalization techniques [9], yet they remain largely ineffective in the sparse Terahertz domain, where the lack of high-fidelity textural landmarks prevents standard alignment. Furthermore, they do not fully exploit the information encoded in the Sub-MMW signal, which contains more than just topographic data [10].

For example, recent work has demonstrated that this latent information includes complementary data from both the amplitude and phase of the multi-spectral THz signal; low-frequency phase can provide precise depth information while the corresponding amplitude can delineate finer object contours, with their fusion leading to improved image restoration [11]. Further expanding on the concept of latent biometric markers, another research has demonstrated that unique and discriminative information is also present in the radiometric signature of the human thorax, with multi linear analysis of this region in MMW images, showing more promising identification results than the facial region alone [12]. Complementing these holistic, texture-based methods, alternative feature-based approaches have also been explored, where a small set of discriminative, distance-based features is extracted from the geometry of the body silhouette, with measures like height and waist width proving effective for user separation [13]. This principle of analyzing unique signal responses has also been applied to fingerprint liveness detection, where the distinctive back-reflection from the inner layers of the epidermis, observable in the THz time-domain signal, provides a reliable way to distinguish real fingers from artificial spoofs [14].

### Applying Deep Learning Approaches for Terahertz and Sub-Millimeter-Wave Imaging

Deep learning offers a promising alternative, with the potential to learn robust, discriminative features directly from the noisy, low-resolution data. Previous work has explored deep learning for specific, isolated tasks like face verification or posture estimation [10,15]. However, a more holistic approach is needed to fully realize the potential of Sub-MMW facial analysis for real-world applications. A single system that can simultaneously verify identity, determine the subject’s posture or orientation, and reconstruct a visual likeness for human analysis would represent a significant leap forward.

Unlike template matching, deep learning models can automatically learn discriminative features directly from the raw or minimally processed THz images. This data-driven approach allows them to be inherently more robust to the variations that degrade the performance of hand-crafted feature-based methods [10]. Research has shown success in adapting pre-trained CNNs like AlexNet and VGG-face to extract meaningful features from millimeter-wave body and face images, a technique known as transfer learning [15]. More advanced, specialized architectures are also being developed, such as the Cross-Feature Fusion Transformer YOLO (CFT-YOLO), which is specifically designed to handle the unique challenges of THz images, including low resolution and high noise levels [16]. These modern frameworks enable not just more reliable verification but also more complex tasks, such as the pixel-wise reconstruction of a clear facial image from the occluded THz data [11]. Further innovation in this area is demonstrated by the mmID system, which employs a conditional generative adversarial network (cGAN) to reconstruct high-resolution images of the human body from low-resolution mmWave radar data, enabling more accurate human identification. This system first estimates the spatial spectrum using the Multiple Signal Classification (MUSIC) algorithm and then uses this as input for the cGAN to generate a high-resolution image, which is then used for identification with a CNN-based classifier [17]. Complementing these holistic, texture-based methods, alternative feature-based approaches have also emphasized learning robust feature representations that can generalize from simple to complex scenes [18], an important principle when dealing with the variable quality of THz imagery.

Another critical application is in security, for the detection of concealed objects. The low quality of THz images, characterized by high noise and poor resolution, makes accurate detection challenging. To overcome this, research has shown the effectiveness of a two-step approach: first, preprocessing the THz images to enhance their quality, and then using a deep learning model for detection. One study successfully used Non-Local Mean (NLM) filtering to suppress noise in the images, followed by the YOLOv7 algorithm to detect concealed objects, achieving high recognition accuracy. This highlights the value of combining image processing with advanced detection models like YOLOv7 to create practical systems for security screening [19]. To combat the challenge of limited and low-quality training data, another approach involves generating synthetic active millimeter-wave imaging (AMWI) datasets using depth-based and distance-based simulation methods, which are then used to train a CNN for more accurate concealed object recognition [20]. Furthermore, to enhance real-time concealed object detection in passive Terahertz security images, an improved Single Shot MultiBox Detector (SSD) has been proposed, which replaces the original VGGNet encoder with ResNet-50, incorporates a feature fusion module, and utilizes a hybrid attention mechanism and Focal Loss to achieve a mean average precision of 99.92% at 17 frames per second [21]. To address the challenge of the human body acting as background noise, another effective strategy involves a parallel method where an improved threshold segmentation technique first locates the human body, after which a Faster R-CNN model is used to detect concealed objects, significantly improving both detection speed and accuracy [22].

Shifting focus to image enhancement, a super-resolution reconstruction algorithm based on a multi-scale channel attention (MS-CA) residual network has been proposed to improve Terahertz images for postal security inspection, achieving higher peak signal-to-noise ratio (PSNR) and structural similarity index (SSIM) compared to traditional deep-learning algorithms [23]. Another work in heterogeneous face recognition (HFR) has largely focused on direct feature matching between thermal and visible domains [24], often ignoring the structural guidance that high-fidelity modalities can provide. More recent advances in Multi-Task Learning [25] suggest that auxiliary objectives, such as pose estimation, can regularize feature learning in data-scarce environments. However, the integration of Cross-Modal Knowledge Distillation [26] within a multi-task framework for sub-millimeter wave biometrics remains under-explored.

While prior research has demonstrated the potential of THz and Sub-MMW imaging for isolated tasks such as concealed object detection, image reconstruction, or biometric identification, these efforts have largely treated each problem separately. A greater potential for robust, real-world security surveillance lies in a more holistic approach that synthesizes these objectives and ensures data integrity against geometric distortions [27]. This paper introduces a unified Multi-Task Learning (MTL) framework specifically designed to address this challenge in the context of concealed facial analysis.

**Figure 1:**
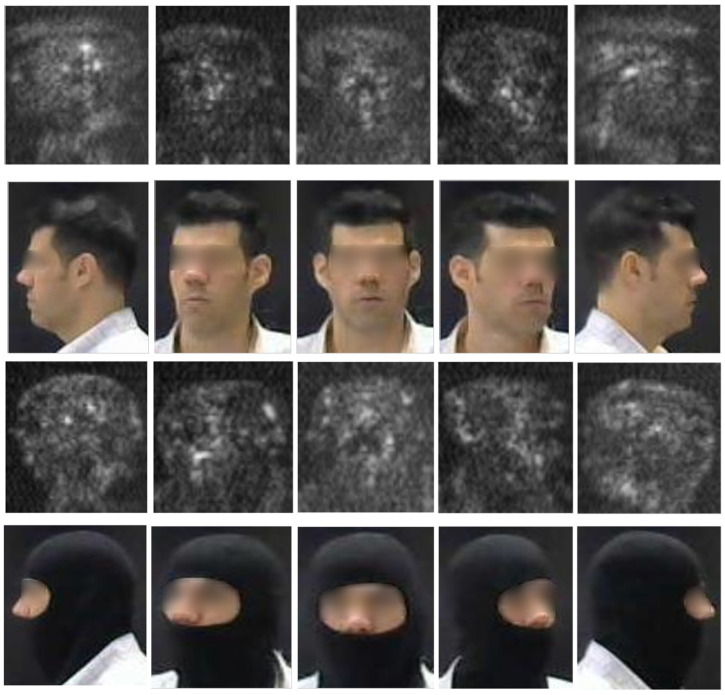
Unconcealed THz images of subject ID #10 across five distinct facial postures, and their visual counterparts. The top two rows present THz unconcealed faces across all facial postures with their corresponding visible images below, while the bottom two rows present the THz concealed faces across all facial postures with their corresponding visible images below. The THz samples demonstrate the inherent challenges of the active 340 GHz modality, characterized by low spatial resolution, high acquisition noise, and sparse geometric representations that obscure fine-grained facial biometrics.

Our primary contributions are as follows: (i) generating, for the first time, reconstructed unconcealed faces from concealed ones (covered by a balaclava), and carrying out facial posture classification in THz images, which are characterized by high noisiness and sparse geometry that obscure fine-grained facial biometrics (as seen in Figure 1). (ii) We propose a pioneering end-to-end deep learning model that simultaneously performs three critical tasks on concealed facial THz images: identity verification, facial posture classification, and unconcealed face reconstruction. (iii) We introduce a novel Cross-Modal Knowledge Distillation model for THz images, where a “privileged” visible-spectrum teacher guides the training of a THz student encoder. By distilling semantic knowledge from the high-fidelity visible domain into the sparse THz latent space, within an MTL framework, our model learns a rich, structure-aware representation of the sparse THz data employed to achieve the three objectives.

The proposed framework moves beyond the limitations of earlier template-matching classical methods and single-focus deep learning methods, presenting a combined novel approach for Sub-MMW facial analysis.

## 2. Materials and Methods

### 2.1. THz Image Acquisition System

The data used in this study were captured using an active frequency-modulated continuous-wave (FMCW) imaging system operating at a center frequency of 340 GHz [5]. The system was configured in a confocal Gregorian optical geometry, designed to acquire head-region intensity images at a nominal standoff distance of approximately 6 m. The transmitter chain consists of Schottky diode multipliers, a pyramidal horn, and a 3 dB high-directivity coupler for transmit-receive coupling. The receiver performs subharmonic mixing to detect the reflected signal. The Sub-MMW beam is focused using a large elliptical reflector and a parabolic sub-reflector. Optical simulations were used to validate the beam propagation, confirming a beam spot of approximately 1.5 cm in diameter at the focus, with a depth of focus of about 41 cm [5]. This optical resolution is sufficient to preserve the macro-geometry of the human face across various facial postures.

To acquire an image, two large galvanometric mirrors mechanically steer the beam across a 45 × 45 cm field of view, scanning at 32 Hz (horizontal) and 1 Hz (vertical). This process yields frames with 64 vertical scan lines, which are then up-sampled to 256 × 64 pixels to maximize the information content. To improve the signal-to-noise ratio (SNR), three consecutive frames, each captured in 0.5 s, are averaged. The final reconstructed images have a dynamic range of approximately 8.3 dB for facial scans. Safety assessments confirmed that the system operates well below the ICNIRP exposure limits for safety, with a peak power density of ~2.0 mW/cm^2^ and an effective exposure of ~2.5 × 10^−4^ s per cm^2^ per frame.

### 2.2. Dataset

The dataset comprises approximately 1400 total Sub-MMW images acquired from 20 different identities [5]. For each identity, images were captured under two conditions: unconcealed (no facial covering) and concealed (wearing a balaclava). To ensure robustness to facial posture variations, data were collected across five distinct face postures: front-left, front-center, front-right, full-left (profile), and full-right (profile). This resulted in approximately 35 unconcealed and 35 concealed images per identity, distributed across the five postures. Figure 1 presents an example of both concealed and unconcealed visible and THz paring face images across five distinct facial postures. For our experiments, a stratified split was performed, holding out 15% of the data for testing. The test set included one concealed and one unconcealed image for each identity in each of the five postures. The remaining data was used for training and validation, with a balanced representation of identities and postures.

In addition to the Sub-MMW image data, our dataset also includes a smaller collection of visible-range images, comprising one concealed and one unconcealed sample for each identity and facial posture. This yields 10 visible-range images per identity, 2 for each of the five facial postures, and a total of 200 images overall. Integrating this auxiliary data enables us to extend the proposed approach into a multi-modal framework, in which knowledge from the visible spectrum can be distilled into the THz domain using a teacher–student training paradigm, while preserving a THz-only configuration at inference time.

All raw images were cropped to the face Region of Interest (ROI). The resulting grayscale ROIs were then standardized to a uniform size of 64 × 64 pixels using bicubic interpolation [28]. This resolution was chosen as a trade-off between preserving facial details and managing the computational complexity of the deep learning model. 

### 2.3. A Unified Multi-Task Learning Using Sub-MMW Imagery Only

To fully utilize the information embedded in Sub-MMW imagery, we developed a novel Multi-Task Learning (MTL) framework. This architecture trains a unified model to simultaneously master three interrelated objectives: concealed face verification, facial posture classification (across both concealed and unconcealed states), and the high-fidelity reconstruction of unconcealed faces from their concealed counterparts. By compelling the network to distill a shared feature representation relevant to all three tasks, our approach enhances data efficiency, improves generalization, and mitigates overfitting, a common challenge in sparse-data domains [25]. The architecture utilizes a shared Convolutional Neural Network (CNN) encoder that projects input Sub-MMW images into a high-dimensional latent space. This mutual representation feeds into three specialized heads: (1) a verification head that generates discriminative embeddings for identity matching, (2) a classification head that predicts discrete head poses, and (3) a reconstruction head that functions as a generative decoder, which generates unconcealed facial images from concealed ones. This joint optimization strategy forces the encoder to disentangle identity-invariant features from pose-dependent variations, yielding a more robust and semantically rich understanding of facial data than is achieved through isolated single-task learning.

In our framework, each training instance is organized in a triplet formulation of anchor–positive–negative structure, that jointly encodes identity information, facial posture variation, and modality asymmetry between concealed and unconcealed facial observations. Specifically, the *anchor* corresponds to a concealed face image of a given identity under a particular facial posture. The *positive* is an unconcealed image of the same identity captured under the same posture. The *negative* is an unconcealed image drawn from a different identity but matched in posture. This structured sampling serves three complementary purposes that enable a unified Multi-Task Learning formulation. First, the anchor–positive pairing provides explicit supervision for concealed-to-unconcealed face verification, requiring the model to extract identity-preserving features despite missing or partially occluded facial information. Second, because all samples are annotated with facial posture labels, the framework naturally supports posture classification, allowing the network to disentangle identity-relevant from pose-dependent cues. Third, the pairing of concealed anchors with their corresponding unconcealed positives provides a reconstruction target that enables unconcealed face synthesis conditioned on concealed inputs, improving both representation separation and robustness across modalities. By feeding all tasks through a shared encoder and by structuring each triplet to reflect identity, pose, and visibility factors, our formulation unifies the three learning objectives under a single sampling.

#### 2.3.1. A Shared Feature Encoding via a Shared CNN Encoder

**Figure 2:**
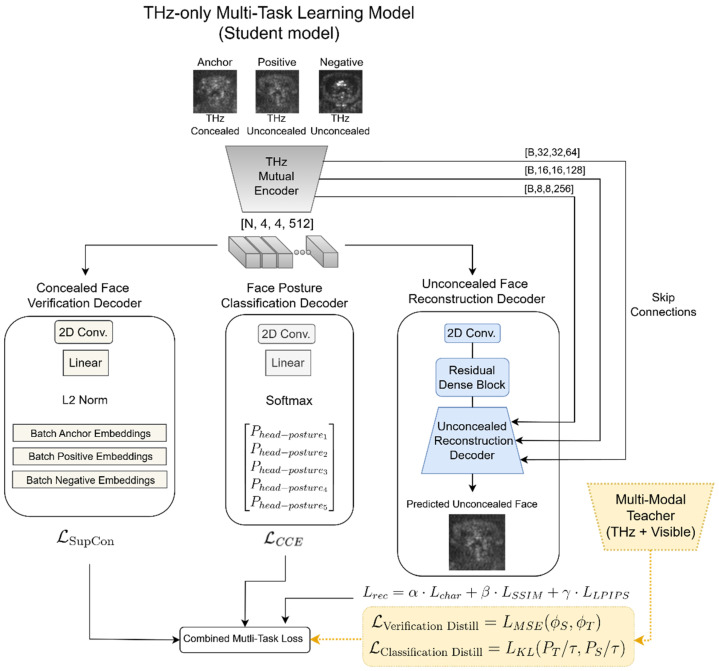
A schematic overview of the THz-only Multi-Task Learning architecture (also, the student model). The model processes a triplet of Sub-MMW facial images (anchor [concealed, subject *i*], positive [unconcealed, subject i], negative [unconcealed, subject j]) through a mutual CNN encoder, which extracts a unified latent representation. This shared feature vector is simultaneously projected into three task-specific branches. The mutual encoder ensures that feature learning is regularized by the competing yet synergistic objectives of all three tasks.

At the core of our MTL framework lies a shared U-Net-type CNN, which serves as the foundational feature encoder for all subsequent tasks, considered as decoders (Figure 2). This shared encoder transforms the input 64 × 64 pixel Sub-MMW facial image into a dense, informative latent representation through a hierarchical series of convolutional layers that progressively abstract low-level textures into high-level semantic features. This architectural strategy, widely validated in multi-task visual facial analysis [24], generates a compact, distilled feature vector that serves as a comprehensive basic descriptor of the essential image content. This latent vector is broadcast simultaneously to the verification, classification, and reconstruction heads (Figure 2, upper part), ensuring that all tasks rely on shared features, where insights from one task improve the others.

#### 2.3.2. Concealed Face Verification Head

The verification head is tasked with authenticating identity by matching a concealed face (e.g., balaclava-masked) against an unconcealed reference (Figure 2, left column). Leveraging a 128-dimensional shared latent representation from the encoder, this module employs a linear projection followed by L2-normalization to constrain embeddings to a unit hypersphere. This normalization is critical for metric learning, ensuring that similarity is measured solely by angular distance (cosine similarity) rather than vector magnitude, thereby improving robustness to illumination and contrast variations [29].

To learn an identity-invariant representation across concealed and unconcealed facial observations, we adopt a supervised contrastive learning objective (SupCon) [30] that generalizes beyond the classical triplet loss. Each sample in the batch is indexed by n∈1,…,N and is associated with an identity label $y_{n}$. Let $z_{n}^{\prime }$ denote the normalized embedding corresponding to the sample n. Posture and concealment attributes are treated as intra-identity variations and do not define the label space. For any chosen anchor sample with index n, we define a positive set that includes all other samples in the batch that share the same identity label as the anchor

(1) $$P\left(n\right)=\{m\in 1,\ldots ,N\;|\;m\neq n,y_{m}=y_{n}\},$$

and a contrastive set that includes all remaining samples in the batch except the anchor

(2) A(n)={m∈1,…,N | m≠n}.

Thus, P(n) contains same-identity samples (both concealed and unconcealed views across postures), whereas A(n) serves as the denominator of the contrastive Softmax and includes all other samples, spanning both same-identity positives and different-identity negatives. SupCon applies its loss once per anchor index, systematically rotating through all samples in the batch. The supervised contrastive loss for the batch [30] is then expressed as

(3) $$L_{SupCon}=\frac{1}{N}\sum_{i=1}^{N}\left[\frac{-1}{\left|P\left(i\right)\right|}\cdot \sum_{p\in P\left(i\right)}log\frac{exp\left(\mathrm{sim}\left(\tilde{z_{i}},\;\tilde{z_{p}}\right)/\tau \right)}{\sum_{k\in A\left(i\right)}exp\left(\mathrm{sim}\left(\tilde{z_{i}},\;\tilde{z_{p}}\right)/\tau \right)}\right]$$

where $\tilde{z_{i}},\;\tilde{z_{p}}$ and $\tilde{z_{k}}$ are the normalized embeddings corresponding to same-identity samples P(n) and different-identity samples  A(n). The outer summation over N iterates once over every sample in the batch, treating each sample in turn as an anchor. The set P(n) contains all other samples that share the same identity label as the anchor n, and therefore defines the positive pairs used to pull same-identity representations together. The set A(n) contains all remaining samples in the batch and forms all the contrastive pairs used in the denominator. These include both the positives and all different-identity negatives, which push the anchor away from unrelated identities. The inner logarithmic Softmax term compares the cosine similarity (sim) between the anchor and each positive to the similarities between the anchor to all samples in the contrastive pairs. The temperature parameter τ controls the sharpness of this Softmax, with smaller values increasing the emphasis on harder negatives. The normalization by |P(n)| ensures that all anchors contribute equally regardless of how many positive views of the same identity appear in the batch. Together, these components encourage a consistent embedding space in which all concealed and unconcealed views belonging to the same identity are compactly clustered, while representations of different identities remain well separated.

#### 2.3.3. Facial Posture Classification Head

This head is designed for the classification of a facial posture into one of five distinct categories. The architectural design centers on a 2D convolutional layer followed by a linear layer that directly precedes a Softmax activation function. This stage of the network is responsible for transforming the high-dimensional feature vectors, which are learned by the preceding layers, into a probabilistic distribution across the five predefined postural classes.

To train this classification head, a Categorical Cross-Entropy (CCE) loss function, $L_{CCE}$, is employed. This loss function is common in multi-class classification tasks, as it quantifies the dissimilarity between the predicted probability distribution and the ground-truth distribution.

#### 2.3.4. Unconcealed Face Reconstruction Decoder

**Figure 3:**
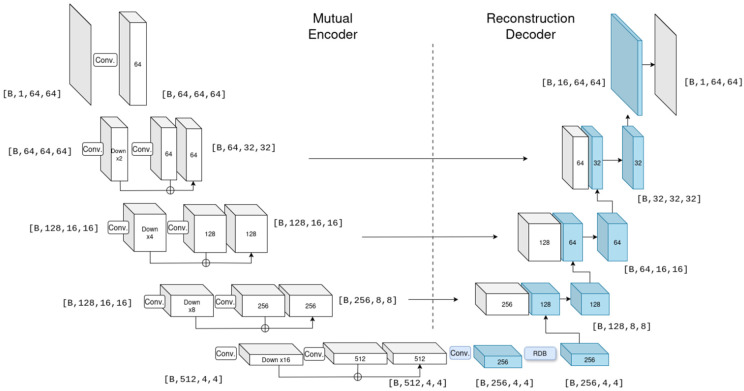
The architecture of the shared CNN encoder (left part, white boxes), combined with the unconcealed face reconstruction decoder (right part, blue boxes). This design is inspired by a U-Net structure, where the encoder uses convolutional layers for learnable down-sampling, and the decoder uses pixel-shuffle layers for up-sampling. A Residual Dense Block (RDB) is placed in the bottleneck for advanced feature extraction.

The primary objective of this decoder is to synthesize the unconcealed facial image from latent representations derived from concealed Sub-MMW sensor data. The architecture functions as a generative decoder, taking a compressed feature vector from the encoder and progressively up-samples it through a sequence of convolutional layers to produce a 64 × 64 pixel resolution image. The reconstruction decoder utilizes a U-Net-like architecture, processing multi-scale encoder features via a bottleneck Residual Dense Block and progressively recovering spatial resolution through PixelShuffle upsampling layers interleaved with skip connections and convolutional fusion blocks, terminating in a sigmoid-activated reconstruction [31]. To enhance the reconstruction fidelity and preserve fine facial details, this decoder incorporates a Residual Dense Block (RDB) as the bottleneck module between the encoder and the decoder parts (right part, Figure 3), which utilizes densely connected convolutional layers where each layer receives feature maps from all preceding layers, enabling efficient feature reuse and gradient flow [31]. The RDB implements local feature fusion via 1 × 1 convolutions to stabilize training, followed by residual connections for hierarchical feature learning. This dense connectivity and residual architecture facilitate robust feature propagation throughout the decoder network, preserving fine-grained facial characteristics during the reconstruction process [31]. The composite loss, $L_{rec}$, is formulated as follows:

(4) $$L_{rec}=\alpha \cdot L_{Charbonnier}\left(x,\hat{x}\right)+\beta \cdot \left(1-\mathrm{SSIM}\left(x,\hat{x}\right)\right)+\gamma \cdot L_{LPIPS}\left(x,\hat{x}\right).$$

The reconstruction loss $L_{rec}$ combines a robust Charbonnier term for pixel-wise fidelity, a (1−SSIM) term, and is the $L_{SSIM}$ for preserving structural similarity, and the $L_{LPIPS}$ term serves for perceptual consistency between x (ground-truth) and $\hat{x}$(predicted) unconcealed THz faces, weighted by α, β, and γ, respectively. Together, these terms jointly enforce low-level accuracy, structural integrity, and high-level perceptual quality in the reconstructed images. The Charbonnier loss component enforces pixel-level fidelity between the reconstructed and ground-truth images. It is a robust variant of the L1 loss that is less sensitive to outliers, ensuring precise pixel-wise correspondence. Its explicit expression is

(5) $$L_{\mathrm{Charbonnier}}=\frac{1}{N}\sum_{i=1}^{N}\sqrt{{\left(I_{Pred}^{\left(i\right)}-I_{\mathrm{GT}}^{\left(i\right)}\right)}^{2}+\epsilon^{2}}.$$

This calculates the average error over all pixels by summing the square-rooted, squared differences between each reconstructed pixel and its ground-truth counterpart, with a small constant ϵ to ensure differentiability and stability. The Structural Similarity Index Measure (SSIM) loss component aims to preserve the local structural similarity between the images, ensuring that textures and fine details are realistically rendered. SSIM evaluates luminance, contrast, and structure. The loss is formulated as 1−SSIM to be minimized. The SSIM index between two images, *x* and *y*, is as follows:

(6) $$SSIM\left(x,y\right)=\frac{\left(2\mu_{x}\mu_{y}+C_{1}\right)\left(2\sigma_{xy}+C_{2}\right)}{\left(\mu_{x}^{2}+\mu_{y}^{2}+C_{1}\right)\left(\sigma_{x}^{2}+\sigma_{y}^{2}+C_{2}\right)}.$$

Here, μ and σ represent the local means and standard deviations, $\sigma_{xy}$ is the cross-covariance, and $C_{1}$ and $C_{2}$ are small constants that stabilize the division. The Learned Perceptual Image Patch Similarity (LPIPS) loss leverages a pre-trained deep neural network to measure the perceptual similarity between the reconstructed and ground-truth images. By comparing feature maps from various layers of a network trained on a large-scale image dataset, LPIPS provides a metric that aligns more closely with human perception of image quality [32]. This promotes the generation of visually plausible and realistic results. The loss is computed as follows:

(7) $$L_{LPIPS}\left(X,X_{0}\right)=\sum_{l}\frac{w_{l}\cdot 1}{H_{l}W_{l}}\sum_{h,w}{\left|y_{h,w}^{l\prime }-y_{0,h,w}^{l\prime }\right|}_{2}^{2}$$

In this equation, $y^{l}$ and $y_{0}^{l}$ are the channel-normalized feature activations from layer l for the two images. The squared L2 distance is computed for each spatial location, averaged, and then combined as a weighted sum across layers, with weights $w_{l}$.

### 2.4. Integration of a Visible-Range Modality: A Teacher–Student Model

Although the proposed multi-task model operates on THz Sub-MMW images only at inference, we introduce a visible–THz learning multi-modal framework that exploits visible-range facial images only during training (Figure 3). The goal is to examine how the THz encoder can benefit from the richer structural cues present in visible images, without requiring an additional sensor at deployment [33,34].

#### 2.4.1. Dual-Encoder Architecture

**Figure 4:**
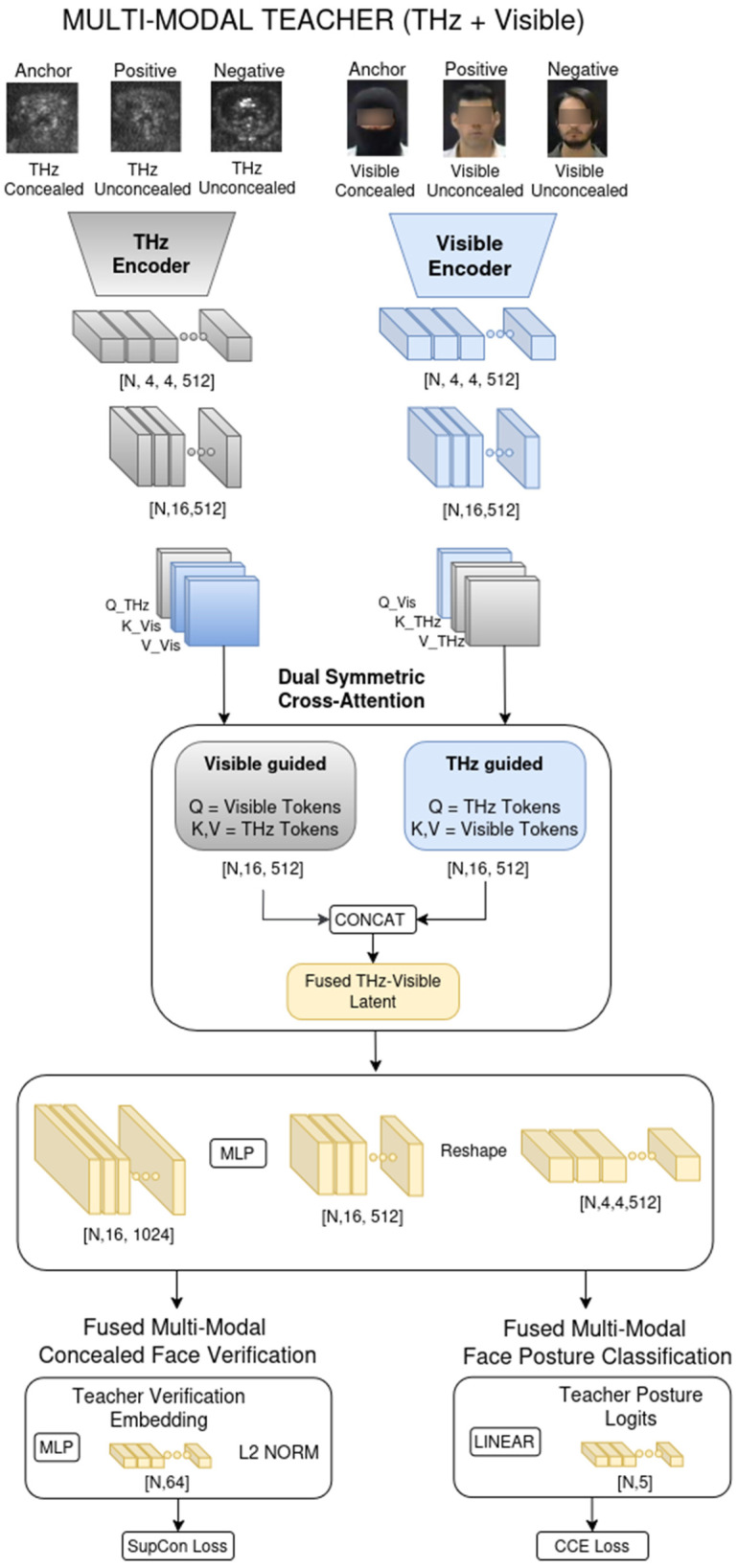
Multi-modal teacher architecture. The framework employs a dual-encoder design where visible and THz encoders process input modalities in parallel. A dual cross-attention module symmetrically fuses features, allowing each modality to query complementary information from the other. The resulting tokens are concatenated and projected into a unified teacher embedding, which drives task-specific heads for identity verification and facial posture classification. This fused representation serves as the semantic target for student distillation.

Our framework adopts a dual-encoder design to enable effective cross-modal knowledge transfer during training [35]. The THz encoder $E_{thz}$ operates on both concealed and unconcealed THz facial images and serves as the sole feature extractor at inference time (Figure 4). Complementing this, a visible-range encoder $E_{vis}$ processes both masked and unmasked visible-spectrum images, but is utilized only during training to provide auxiliary supervisory signals. Each modality’s encoder (Figure 4) produces a spatial feature representation that is subsequently projected into a sequence of latent tokens $T_{thz},T_{vis}$, forming the basis for the multi-modal knowledge distillation process, a strategy akin to the cross-modal teacher-student frameworks proposed by [36].

#### 2.4.2. Cross-Modal Fusion via Symmetric Cross-Attention

To robustly transfer semantic structure from the visible domain into the THz feature space, we employ a dual cross-attention mechanism that symmetrically fuses features guided by each modality [37]. This module consists of two parallel attention branches: a visible-guided branch where visible tokens act as queries to extract relevant details from the THz context, and a THz-guided branch where THz tokens query the visible features to hallucinate missing high-frequency textures [34].

Let $T_{vis}$ and $T_{thz}$ denote the token sequences from the visible and THz encoders, respectively. The visible-guided cross-attention is computed as follows:

(8) $$\begin{array}{c} Q_{vis}=T_{vis}W_{Q}^{vis},K_{thz}=T_{thz}W_{K}^{vis},V_{thz}=T_{thz}W_{V}^{vis}\\ T_{vis\to thz}=Softmax\left(\frac{Q_{vis}K_{thz}^{⊤}}{\sqrt{d_{k}}}\right)\cdot V_{thz} \end{array}$$

where $Q_{vis}$ projects the visible-domain token features $T_{vis}$ into a query embedding space using a learnable visible-query projection matrix, $W_{Q}^{vis}$ to produce visible queries. $K_{thz}$ projects the THz-domain token features $T_{thz}$ into a key embedding space using a projection matrix $W_{K}^{vis}$ (shared with or aligned to the visible domain) to obtain cross-modal keys $K_{thz}$. $V_{thz}$ projects the THz-domain token features $T_{thz}$ into a value embedding space via $W_{V}^{vis}$, producing cross-modal values $V_{thz}$ that carry THz content information. $T_{vis\to thz}$ computes cross-attention from visible queries to THz keys, normalized by $\sqrt{d_{k}}$ and passed through a Softmax, then aggregates THz values $V_{thz}$ to produce THz-aware visible features $T_{vis\to thz}$. Symmetrically, the THz-guided cross-attention is defined as the following:

(9) $$\begin{array}{c} Q_{thz}=T_{thz}W_{Q}^{thz},K_{vis}=T_{vis}W_{K}^{thz},V_{vis}=T_{vis}W_{V}^{thz}\\ T_{thz\to vis}=Softmax\left(\frac{Q_{thz}K_{vis}^{⊤}}{\sqrt{d_{k}}}\right)\cdot V_{vis} \end{array}$$

The final fused representation is obtained by concatenating these cross-attention features and projecting them back to the original dimension via a Multi-Layer Perceptron (MLP), yielding a unified teacher embedding for distillation [37]:

(10) $$f_{fused}=MLP\left(\left[T_{vis}\to thz∥T_{thz}\to vis\right]\right)$$

This fused representation effectively combines the semantic robustness of the visible domain with the modality-specific geometry of the THz domain, serving as a comprehensive target for the student encoder [38].

#### 2.4.3. Teacher–Student Knowledge Distillation

To ensure that the final deployed system operates exclusively on the THz dataset, we incorporate a teacher–student distillation framework. The teacher branch leverages the fused THz–visible embedding $f_{fused}$, along with its task-specific heads for identity verification and head-posture prediction (Figure 3). In parallel, the student branch relies solely on the THz-derived embedding $f_{thz}$, extracted from the THz encoder $E_{thz}$, and uses analogous prediction heads. During training, knowledge is transferred from teacher to student through two complementary distillation objectives, enabling the THz-only student model to learn semantic structure and discriminative capability from the multi-modal teacher model [26].

At the representation level, the student embedding $f_{thz}$ is projected into a shared latent space and trained to match the teacher’s fused embedding [39]. This is formulated as

(11) $$f_{fused}=MLP\left(\left[T_{vis}\to thz∥T_{thz}\to vis\right]\right)$$

(12) $$L_{\mathrm{feature\_distillation}}=\frac{1}{N}\sum_{i=1}^{N}{\left(\phi_{S}{\left(f_{thz}\right)}_{i}-\phi_{T}{\left(f_{fused}\right)}_{i}\right)}^{2}.$$

The cost function computes the squared L2 distance between student and teacher embeddings. $f_{thz}$ is the latent feature map produced by the THz encoder $E_{thz}$. $\phi_{S}$ is a student projection head from the encoder output into an embedding vector [40]. $f_{fused}$ is the multi-modal fused latent feature map created by integrating THz and visible features via cross-attention (Equation (10)), and $\phi_{T}$ is the teacher projection head, producing the target representation.

The feature-level distillation objective encourages the THz encoder to absorb semantic structure derived from the visible modality, including identity-discriminative cues, posture-related information, and both global and local relational patterns encoded in the fused representation [24]. Since the teacher integrates multi-modal inputs, its fused embeddings provide a richer and more informative target than THz-only features. By aligning with this target, the student effectively approximates modality-specific information [41] unavailable in the THz domain, thereby enhancing its representational capacity while remaining single modality at inference.

For posture classification, we employ a logit-level distillation objective in which the student is supervised by the teacher’s softened output distribution [42]. The loss is defined as

(13) $$L_{\mathrm{logit\_distillation}}=\tau^{2}\cdot KL\left(\phi_{T}∥\phi_{S}\right)$$

(14) $$KL\left(\phi_{T}∥\phi_{S}\right)=\sum_{i}softmax\left(p_{T,i}/\tau \right)log\left(\frac{softmax\left(p_{T,i}/\tau \right)}{softmax\left(p_{S,i}/\tau \right)}\right)$$

where $p_{T}$ and $p_{S}$ denote the teacher and student logits, respectively, and τ is a temperature parameter that smooths the probability distributions. $p_{T}$ are the raw, pre-Softmax outputs of the teacher classifier, that encode the teacher’s confidence for each posture class before normalization. $p_{S}$ are the corresponding raw outputs of the student (THz-only) classifier. τ is the temperature scaling parameter which smooths the probability vector distribution for a value greater than 1.0 [42]. Both teacher and student logits are passed through a Softmax, with the temperature scaling parameter. The KL divergence (Equation (14)) measures how different the student’s softened distribution is from the teacher’s. This formulation provides richer inter-class similarity information than hard labels, allowing the THz-only classifier to approximate the more informative and discriminative decision boundaries learned by the fused multi-modal teacher. The $\tau^{2}$ scaling factor is a standard correction factor in temperature-based distillation. It preserves gradient magnitudes when increasing the temperature, otherwise raising τ would artificially shrink gradients, weakening training. By matching the teacher’s softened logits, the student learns fine-grained inter-class relationships and smoother decision boundaries, while implicitly absorbing multi-modal cues present in the fused teacher representation. This enables the THz-only classifier to benefit from the behavior of the more expressive multi-modal teacher during inference.

**Figure 5:**
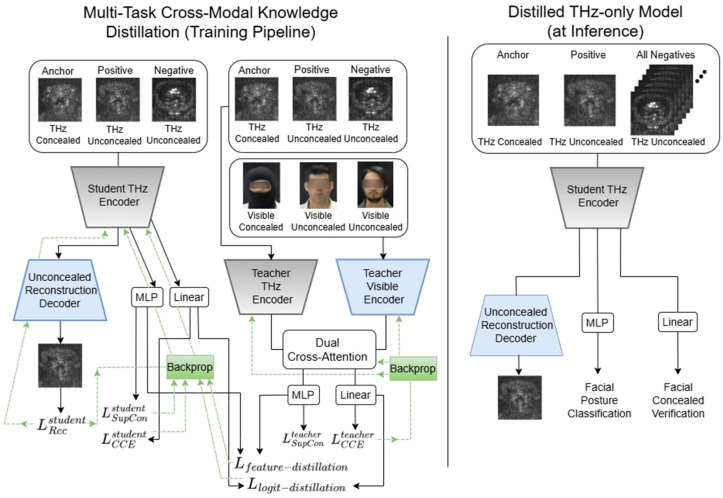
Training and inference pipelines in the cross-modal teacher–student knowledge distillation framework. During training (**left part**), student and teacher models are optimized jointly through knowledge distillation and their own loss terms. The green dashed lines indicate the back-propagation direction for each loss term. Distillation losses are computed between the verification embeddings and classification logits of the teacher and student networks, and are used to optimize the student model parameters. At inference (**right part**), the teacher is discarded, and predictions are generated using only the student model with THz input data.

During training, the model processes fully paired multi-modal inputs: a THz triplet (concealed anchor, unconcealed positive, unconcealed negative) is fed into the THz encoder, while a corresponding visible triplet (concealed anchor, unconcealed positive, unconcealed negative) is processed by the visible encoder. These parallel feature streams are integrated via a symmetric dual cross-attention mechanism, where the visible triplet queries the THz features for non-visual geometric context, and the THz triplet queries the visible features to hallucinate missing textural details. Crucially, the visible stream serves strictly as privileged information [43]. Once training is complete, the entire teacher pathway that comprises the visible encoder, the cross-attention fusion module, and all teacher-specific prediction heads is discarded. At inference, only the THz encoder and the student’s verification and head-posture classification are retained. This ensures that the deployed system maintains the computational efficiency of a single-modality THz model while leveraging the robust, structure-aware representations distilled from the multi-modal teacher. The multi-modal teacher–student training framework versus the THz-only model at inference is described in Figure 5.

## 3. Results

The dataset was prepared from a total of approximately 1400 images. We employed only samples where, for a given subject and facial posture, both concealed and unconcealed images were available. This was necessary for the triplet-based training methodology, which requires anchor (concealed) and positive (unconcealed) pairs. This selection process resulted in a usable dataset of 1158 images.

This dataset was then divided into training/validation and testing sets using a sterile, stratified methodology. This approach guarantees that there is no overlap of a subject’s specific image samples between the sets, preventing any form of data leakage that could inflate performance metrics. The data division was performed based on both the subject’s identity and the specific facial posture, ensuring that all variations were proportionally represented. For testing, at least one concealed sample per identity per posture was kept aside as a sterile hold-out set. Similarly, validation folds maintained separation by identity and posture.

### 3.1. Performance Results of the THz-Only and the Distilled Multi-Modal Models

To produce reliable test results given a limited database size, we conducted an all-folds-as-test cross-validation (CV) study and present. The average results of all test folds are shown in Table 1, including concealed face verification accuracy, posture classification accuracy, and quantitative measures of reconstruction of unconcealed facial images from concealed ones. The dataset is divided into 6 folds. For each run, one fold serves as the test set, one as the validation set, and the remaining four as training data. This protocol ensures that every triplet in the dataset (N = 238,958) is evaluated as test data exactly once across the 6 runs. Both Model A (THz-only) and Model B (distilled multi-modal) are trained and evaluated under identical conditions for each fold.

Both models exhibit low cross-fold variance across all metrics, confirming that performance is stable regardless of the data partition. Comparison across the 6 folds reveals that Model B achieves a wider L2 distance margin than Model A (+0.032, 95% CI [+0.008, +0.056], *p* < 0.05) and lower cosine positive distance (0.027 vs. 0.045, 95% CI for delta: [−0.025, −0.011]), indicating that the multi-modal teacher produces more discriminative embeddings. Model A achieves higher reconstruction quality (windowed SSIM 0.615 vs. 0.559, PSNR 25.50 vs. 25.14 dB; both at *p* < 0.05). Per-identity analysis confirms that all 20 identities exceed 94% verification accuracy across folds, with 13 of 20 achieving perfect accuracy (100%, std = 0).

**Figure 6:**
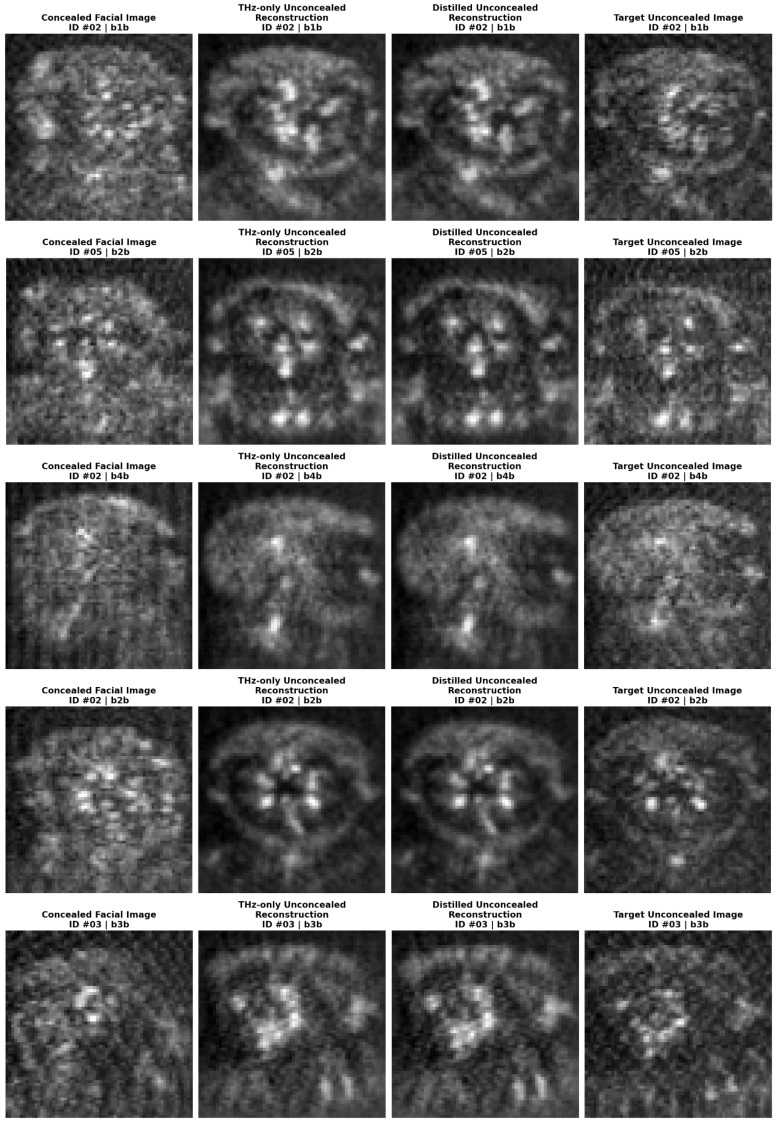
A visual comparison of unconcealed face reconstruction from concealed THz images for different identities and postures. From left to right: concealed facial input images, THz-only model reconstructions, distilled model reconstructions, and ground truth unconcealed target images. Visual inspection reveals comparable reconstruction fidelity between the two models. Subject identifiers and posture codes are indicated above each image.

Qualitative visual results of unconcealed THz face reconstruction from concealed THz images for different identities and postures are shown in Figure 6. Inspection of Figure 6 offers further insights beyond the numerical reconstruction fidelity. Visually, the reconstructed unconcealed THz images from both models appear robust, successfully preserving key facial structures and biometric patterns inherent to the subjects. The generated output exhibits a smooth, denoised appearance. This smoothing results in coherent facial representations that allow for clearer interpretation of facial morphology, suggesting that the perceptual quality of the reconstruction may exceed what strict pixel-level metrics like SSIM and PSNR imply. This performance parity is significant as it demonstrates that the distillation process did not compromise the generative capabilities of the network. Typically, optimizing an embedding space for a discriminative task (verification), risks “feature collapse” [44] where the fine-grained textural information needed for reconstruction is discarded in favor of abstract, identity-focused representations. However, the results indicate that the multi-modal distillation effectively regularized the latent space without such degradation.

### 3.2. Open-Set Generalization Analysis

To evaluate the model’s ability to generalize entirely from unseen identities, we conducted a Leave-Two-Subjects-Out (LTSO) cross-validation study (Table 2). The 20 identities are partitioned into 10 non-overlapping pairs. In each fold, one pair (2 identities) is held out for testing, and the remaining 18 identities are used for training (85%) and validation (15%). Over 10 folds, every identity serves as held-out test exactly once, yielding 11,816 test triplets in total. Both THz-only model and distilled multi-modal model are trained and evaluated under identical conditions.

Verification accuracy drops from ~99% under data-disjoint cross-validation (Table 1) to 75.24% for the THz-only model and 73.42% for the distilled multi-modal model under LTSO, confirming that identity verification on completely unseen subjects is harder. This is an expected result when the model must generalize from only 18 training identities. Both models remain well above the 50% random baseline for triplet verification, demonstrating open-set generalization. Reconstruction quality is also reduced to windowed SSIM ~0.30, PSNR ~21.4 dB from windowed SSIM ~0.60, PSNR ~25.0 dB, showing dependency on the individual identity. Posture classification accuracy (96%) remains robust, as it does not depend on individual identity and transfers effectively across subjects. High inter-fold variance (std 10–15%) reflects identity-dependent difficulty; verification accuracy ranges from 64% to 94% (THz-only) and 47% to 96% (distilled multi-modal) across folds. Paired comparison across the 10 folds reveals no statistically significant difference in verification accuracy between the two models (delta = −1.83 pp, 95% CI [−11.8, +8.1]). Both architectures perform quite similarly when tested on unseen identities. The multi-modal model achieves lower cosine positive distance (0.307 vs. 0.369, delta = −0.062, 95% CI [−0.104, −0.020]) and significantly lower L2 distances, indicating that cross-modal distillation produces tighter embedding clusters even under subject-disjoint evaluation. Per-identity analysis shows that verification accuracy ranges from 32% (hardest identity) to 100% (easiest) for the THz-only, and from 25% to 100% for the multi-modal, reflecting the inherent challenge of open-set recognition with a 20-identity training pool.

### 3.3. Single-Task vs. Multi-Task Learning Ablation

To quantify the performance gains that are due to the Multi-Task Learning (MTL) framework, we conducted a systematic ablation study comparing all possible task combinations: three single-task (STL) configurations (Verification-only [V], Classification-only [C], Reconstruction-only [R]), three dual-task configurations (V + C, V + R, C + R), and the full three-task MTL configuration (V + C + R). All seven variants share an identical architecture and hyperparameters (backbone, embedding dimension, learning rate, augmentation). Only the active loss terms differ. Each variant was evaluated on the sterile test set (N = 2052 triplets) with no subject-level overlap with the training data. Results are shown in Table 3.

The full MTL framework achieves the highest performance across all three tasks simultaneously. Verification accuracy improves progressively with task co-training: V-only (99.12%) to V + R (99.17%) to V + C + R (99.81%), a relative error reduction of 78% over the STL baseline. The verification distance margin increases correspondingly from 0.964 to 0.999 to 1.028, indicating that the shared encoder learns increasingly discriminative embeddings when regularized by multiple objectives. Classification reaches perfect accuracy (100%) in both V + C and V + C + R configurations, compared to 99.38% for the C-only baseline, suggesting that the verification objective provides complementary supervision that refines posture decision boundaries. Reconstruction quality is preserved across all configurations where it is trained; windowed SSIM remains stable (R: 0.482, V + R: 0.482, C + R: 0.480, V + C + R: 0.485) with PSNR marginally improving from 24.29 dB to 24.36 dB in the full MTL setting, confirming that discriminative task gradients do not cause feature collapse in the generative pathway.

### 3.4. Knowledge Distillation Loss-Weight Analysis

To investigate the effect of knowledge distillation (KD) configuration on embedding quality, we conducted a 6-variant ablation study examining four aspects. The first is component isolation (feature vs. logit distillation), the second is weight scaling, the third is asymmetric weighting, and the fourth is the temperature parameter. Component isolation disables one KD channel to measure its individual contribution; weight scaling uniformly increases or decreases both distillation losses to test for saturation; asymmetric weighting shifts the balance toward feature or logit distillation to identify the dominant driver, and temperature varies the Softmax sharpness of the logit target distribution. 

In the results shown in Table 4, all variants share an identical architecture and hyperparameters, differing only in the KD loss weights and temperature. Each variant is evaluated on the sterile test set (N = 2052 triplets) with no subject-level overlap with training data.

The default KD configuration (feat_w = 1.0, logit_w = 0.5, T = 3.0) reduces intra-class positive distance by 7.0% (0.2276 to 0.2116) while simultaneously increasing the inter-class margin by 1.3% (0.9676 to 0.9800) and the normalized margin by 4.2% (2.355 to 2.454) relative to the no-KD baseline, demonstrating that distillation improves both compactness and separability without trade-off. Both KD components independently contribute; feature-only distillation reduces positive distance by 4.2% and logit-only by 4.5%, with the combined configuration achieving 7.0%, indicating approximately additive benefits. The logit-heavy variant (feat_w = 0.3, logit_w = 1.0) achieves the best compactness (0.2071, −9.0% vs. no_KD), margin (0.9858, +1.9%), and verification accuracy (99.81%), suggesting that soft label transfer via KL-divergence on temperature-scaled logits provides a stronger implicit compactness constraint than L2 feature matching by encoding inter-class relational structure. The feature-heavy variant (feat_w = 2.0, logit_w = 0.15) yields the least compactness (0.2238, −1.7% vs. no_KD) and lowest reconstruction quality (W-SSIM = 0.462, −1.1%), despite maintaining competitive verification accuracy (99.66%), indicating that over-emphasis on explicit L2 feature alignment without sufficient logit-based relational guidance leads to degraded feature distribution and visual fidelity. Reconstruction quality remains stable across all variants (W-SSIM 0.467–0.471, PSNR 24.17–24.27 dB), confirming that KD configuration does not affect the generative pathway.

### 3.5. Unconcealed Facial Reconstruction Fidelity

To validate that the reconstruction pathway performs genuine occlusion-invariant reconstruction rather than hallucinated generation from memorized priors, we conducted a controlled 2-variant study. We compare two training variants with identical decoder architectures: (i) the proposed THz-only model that feeds concealed anchor features to reconstruct unconcealed positive THz images (learning occlusion-invariant mapping), and (ii) a similar THz-only model (‘Autoencoder’) that feeds unconcealed positive features to reconstruct the same image (learning near-identity mapping). Both variants are evaluated under concealed and unconcealed input conditions, creating four test configurations that isolate occlusion-invariant reconstruction ability, cross-condition generalization, the impact of training without occlusion signals, and the Autoencoder architectural capacity. Beyond standard pixel-level metrics (W-SSIM, PSNR), we report metrics considered perception-related (Table 5): Edge SSIM (Sobel gradient boundary preservation) [45], Edge Mean Absolute Error (edges mean absolute gradient error), embedding cosine similarity (deep identity-level preservation via global-average-pooled encoder features), and an anatomically grounded metric of regional SSIM across five facial regions (Table 6).

The proposed model outperforms the Autoencoder model on concealed reconstruction: W-SSIM 0.468 vs. 0.216 (+116.9%), PSNR 24.21 vs. 19.93 dB (+4.28 dB), confirming genuine occlusion-invariant learning rather than a memorized generation. The baseline’s near-perfect performance on unconcealed inputs and outputs (W-SSIM 0.991, PSNR 41.43 dB) validates that the architecture has full reconstruction capacity and that its collapse on concealed inputs (W-SSIM 0.216) is due exclusively to the absence of occlusion-invariant training signals, not architectural limitations. Perception-related metrics confirm structural fidelity beyond pixel-level similarity: Edge SSIM +101.1% (0.223 vs. 0.111) and embedding cosine similarity +4.0% (0.965 vs. 0.928). Regional SSIM analysis across five facial regions (Table 6) reveals that the proposed model achieves the largest improvements in the most heavily occluded areas: the center/nose region improves from 0.173 to 0.483 (+179%), the mouth from 0.185 to 0.449 (+143%), and the periphery from 0.230 to 0.495 (+115%), while the eye regions show more moderate gains (left eye: 0.270 to 0.537, +99%; right eye: 0.265 to 0.515, +94%). This pattern demonstrates that the model learns to reconstruct occluded facial anatomy rather than merely reproducing surface-level patterns, with reconstruction quality correlating inversely with occlusion severity.

### 3.6. Cross-Attention Fusion Study

To validate the necessity of the proposed symmetric cross-attention fusion, we conducted a 4-variant ablation study comparing: (i) no fusion (per-token concatenation with linear projection), (ii) visible-guided cross-attention only (Q = Visible, K/V = THz), (iii) THz-guided cross-attention only (Q = THz, K,V = Visible), and (iv) the proposed dual cross-attention (symmetric bidirectional). All variants share an identical backbone architecture and hyperparameters, differing only in the fusion mechanism. Each variant is evaluated on the sterile test set with no subject-level overlap with training data. Results are shown in Table 7.

The visible-guided variant (99.56%) outperforms both the no-fusion baseline (99.32%) and the THz-guided variant (98.98%) in verification accuracy. This confirms that cross-attention fusion produces higher-quality teacher representations than simple feature concatenation, and that the visible-guided direction (Q = Vis, K/V = THz) is the critical pathway: letting visible features attend to THz features is more informative than the reverse. The dual cross-attention variant achieves the best reconstruction fidelity (W-SSIM 0.469, SSIM 0.780), indicating that the THz-guided branch contributes complementary spatial information that benefits the generative reconstruction decoder. All four variants achieve verification accuracy above 98.9% and identical 99.38% classification accuracy, demonstrating that the framework is robust to the choice of fusion strategy. The proposed dual design represents a balanced trade-off: it achieves the best reconstruction quality while maintaining competitive verification performance (99.27%), confirming it as the appropriate choice for the joint multi-task objective that includes both discriminative and generative tasks.

### 3.7. Cross-Modal Distillation Semantic Transfer

To address whether the L2 feature distillation loss (Equation (12)) produces semantically meaningful alignment or a numerically close but representationally degenerate mapping, we conducted a 4-variant distillation study (Table 8). All variants share an identical architecture and hyperparameters differing only in how the distillation signal is configured: (i) proposed (full model), (ii) no distillation (feature and logit distillation weights set to 0), (iii) random teacher (teacher frozen at random initialization), and (iv) permuted distillation (feature distillation targets shuffled across the batch dimension, destroying sample-wise correspondence while preserving distribution statistics). Each variant is evaluated on the sterile test set with no subject-level overlap with training data. Alignment metrics are measured in two representation spaces: the projection space, where Equation (12) directly operates, and the embedding space, where downstream task heads (verification, classification) consume representations to distinguish numerical proximity from semantic transfer.

High alignment here means distillation knowledge transfers beyond the projection bottleneck to task-relevant features. A model showing high projection alignment but low embedding alignment has learned a numerically close but semantically empty mapping. Three alignment metrics are reported in each space. Linear CKA (Centered Kernel Alignment) measures whether two spaces encode the same relational geometry, whether samples that are similar in one space are also similar in the other (1.0 = identical structure, 0 = unrelated). Spearman *ρ* is a rank correlation over all pairwise distances, testing whether the global distance topology is preserved. Cosine similarity measures the average directional alignment of individual feature vectors. High cosine with low CKA indicates distribution-level matching without sample-specific structure. Retrieval Rank-5 checks whether each identity’s student centroid finds the correct identity among the 5 nearest teacher centroids, the most direct test of identity-level semantic preservation.

Three findings emerge. First, distillation creates genuine structural alignment beyond shared-encoder effects: the proposed model’s projection-space CKA (0.839) exceeds the no-distillation model (0.558) by 50%, with Spearman *ρ* nearly doubling (0.343 to 0.634), confirming that Equation (12) actively shapes the student’s projection space to match the teacher’s representational geometry. Second, the random teacher variant reveals that numerical alignment alone is insufficient for semantic transfer: despite achieving the highest projection-space CKA (0.927) and Spearman *ρ* (0.917), the student successfully copies the frozen random teacher’s projections, the projection vs embedding columns of Table 8 show this numerically excellent alignment carries no semantic content, as the random teacher has the worst embedding-space CKA (0.467†), worst Spearman *ρ* (0.335†), and worst cross-modal retrieval (Ret@5: 10%† vs 30% for proposed). Meaningful contribution from the random teacher has not been transferred to the student. The student succeeds via task losses alone (99.56% Ver. Acc), not because of distillation. This dissociation between numerical proximity and semantic transfer demonstrates precisely the degenerate mapping failure mode, and confirms that teacher quality, and not loss minimization alone, determines whether alignment carries meaningful cross-modal knowledge. Third, permuted distillation shows high cosine similarity (0.922, nearly matching proposed at 0.948) but collapsed CKA (0.480) and Spearman *ρ* (0.330), confirming that Equation (12) captures sample-level identity structure rather than distribution-level statistics: permutation preserves the marginal distribution of teacher projections (enabling high average directional alignment) while destroying the identity-specific correspondence that CKA and distance topology metrics detect. All four variants achieve verification accuracy above 98.67% with the teacher pathway entirely removed at inference, confirming the student retains full THz-only capability regardless of distillation configuration.

## 4. Discussion

This study presents a unified Multi-Task Learning (MTL) framework for concealed face analysis in the THz domain and evaluates its behavior through a series of controlled experiments.

Cross-validation across all six folds (Section 3.1) confirms that the results of face verification accuracy, posture classification accuracy, and unconcealed THz face reconstruction measures are stable with respect to data partitioning: both models maintain low variance in verification (~99.4 ÷ 99.7%), classification (~99.4 ÷ 99.5%), and reconstruction metrics. The distilled model achieves a wider L2 distance margin (+0.032, *p* < 0.05), while the THz-only model yields higher reconstruction fidelity (W-SSIM 0.615 vs. 0.559, *p* < 0.05). When evaluated in an open-set setting via Leave-Two-Subjects-Out (Section 3.2), verification accuracy drops to ~75%, which is expected given a training pool of only 18 identities per fold. Classification accuracy remains robust at 96%, as posture recognition does not depend on individual identity. Reconstruction quality decreases by roughly 50% (W-SSIM ~0.30 vs. ~0.56–0.62 under closed-set CV), which is consistent with the model encountering entirely unseen facial geometries that were not represented during training. The per-fold variance in open-set verification (std 10÷15%) reflects the inherent difficulty differences among individual identities.

The STL vs. MTL ablation (Section 3.3) shows that joint training across verification, classification, and reconstruction yields consistent improvements over single-task baselines. Reconstruction quality (W-SSIM, PSNR) remains stable across all configurations where it is active, indicating that discriminative and generative objectives do not interfere within the shared encoder.

The KD loss-weight analysis (Section 3.4) indicates that both feature-level and logit-level distillation contribute to embedding quality, with approximately additive effects on intra-class compactness and inter-class margin. The logit-heavy configuration produced the largest compactness gain (−9.0% positive distance vs. no-KD) alongside the highest verification accuracy (99.81%), suggesting that soft-label transfer via temperature-scaled KL-divergence provides an effective implicit compactness constraint. Reconstruction metrics remain unchanged across all KD variants, confirming that the distillation configuration does not affect the generative pathway.

The reconstruction validity study (Section 3.5) addresses whether the decoder performs genuine occlusion-invariant reconstruction or relies on memorized priors. The proposed model outperforms an unconcealed-input model on concealed test inputs (W-SSIM 0.468 vs. 0.216; PSNR 24.21 vs. 19.93 dB), with the largest gains concentrated in the most occluded facial regions (center/nose +194.5% SSIM, mouth +141.6%). The baseline’s collapse on concealed inputs despite near-perfect unconcealed performance (W-SSIM 0.991) indicates that the proposed model’s advantage stems from occlusion-specific learned representations rather than architectural capacity alone. The cross-attention fusion study (Section 3.6) shows that the visible-guided attention direction (Q = Vis, K/V = THz) is the most informative for verification, while the dual symmetric design achieves the best reconstruction fidelity, supporting its use as a balanced choice for the joint multi-task objective.

The distillation integrity study (Section 3.7) provides evidence that the L2 feature distillation loss produces semantically meaningful alignment rather than a numerically close but degenerate mapping. A random teacher control achieves the highest projection-space alignment (CKA 0.927) yet the lowest embedding-space alignment (CKA 0.467) and cross-modal retrieval (Retrieval Rank-5: 10%), demonstrating that numerical proximity alone does not imply semantic transfer. The proposed distillation, by contrast, shows consistent alignment in both projection and embedding spaces, with the highest cross-modal retrieval (30%).

A limitation of this work is the dataset scale of 20 identities. The open-set evaluation (Section 3.2) quantifies this constraint directly, as average verification accuracy on unseen identities reduces to ~75%, with high per-fold variance. Expanding the identity pool and testing under additional real-world factors (distance variation, different concealment materials) remains necessary to assess scalability.

Regarding the image acquisition speed, the system is relatively slow in response (0.5 s; 2 fps). In such a case, motion during image capture that may occur in real-life scenarios can affect the deep learning performances due to possible motion-induced image distortions. However, in such situations, the THz facial image analysis developed here may better benefit systems that can image at faster rates [46]. Future research should apply factors such as significant facial movements, changes with distance, or different camouflage materials.

The use of such a system may raise some privacy issues when the person(s) who occlude their faces are justified in doing so and do not wish for a reconstruction of their non-occluded faces. We believe that such issues can be mitigated using updates to the person’s clothing that identify this individual as a friend, or by purging their images digitally in the database where their images will be stored by comparing friend concealed faces to a database of THz images of non-concealed faces. We also note that a visible reconstruction of the person’s face is not researched here, and MMW images of non-occluded faces do not reveal the actual visible facial structure, but a very sparse representation of the face (as can be seen in Figure 1, comparing the visible and the MMW face images).

## 5. Conclusions

This work establishes a unified multi-task framework that successfully delivers novel objectives in THz facial imaging, which include concealed face verification, facial posture classification, and generative reconstruction of unconcealed faces from concealed faces on a very challenging THz image dataset. We show that robust multi-objective performance is attainable even when relying exclusively on a THz-only dataset. Beyond performance metrics, this study demonstrates that cross-modal distillation can effectively regularize a student model without the need for paired multi-spectral (visible-THz) data at inference time. This may provide a reference for deploying high-fidelity biometric systems in resource-constrained environments, potentially enhancing decision boundaries and uncertainty calibration. Our findings illustrate the feasibility of unified architectures that do not compromise generative interpretability for the sake of discriminative accuracy. The ability of a single multi-task encoder to support both secure verification and human-readable reconstruction suggests a path toward more efficient, privacy-preserving security scanners. Future research can further explore adaptive distillation mechanisms, potentially leading to hybrid systems that blend the structural precision of optical imaging with the unique penetrative capabilities of sub-millimeter waves. The implementation code and trained weights are provided at https://github.com/noamberg/thz-face-distil.

## Figures and Tables

**Table 1:** Cross-validation results: all-folds-as-test, mean +/− std over N = 6 folds, cosine similarity distances are reported. Windowed SSIM uses an 11 × 11 Gaussian kernel.

Metric	THz-Only Model	Distilled Multi-Modal Model
Verification Acc. (%)	99.43 +/− 0.76	99.70 +/− 0.53
Classification Acc. (%)	99.36 +/− 0.69	99.50 +/− 0.72
Avg. Cosine Positive Distance	0.045 +/− 0.007	0.027 +/− 0.009
Avg. Cosine Negative Distance	0.774 +/− 0.004	0.755 +/− 0.010
L2 Distance Margin	0.996 +/− 0.021	1.028 +/− 0.015
Windowed SSIM	0.615 +/− 0.007	0.559 +/− 0.012
PSNR (dB)	25.50 +/− 0.10	25.14 +/− 0.12

**Table 2:** LTSO open-set generalization results (mean +/− std over N = 10 folds, 11,816 total test triplets).

Metric	THz-Only Model	Distilled Multi-Modal Model
Verification Acc. (%)	75.24 +/− 10.52	73.42 +/− 15.45
Classification Acc. (%)	96.13 +/− 5.58	95.88 +/− 3.76
Avg. Cosine Pos. Distance	0.369 +/− 0.115	0.307 +/− 0.121
Avg. Cosine Neg. Distance	0.592 +/− 0.112	0.496 +/− 0.158
L2 Distance Margin	0.245 +/− 0.108	0.223 +/− 0.120
Windowed SSIM	0.301 +/− 0.020	0.300 +/− 0.016
PSNR (dB)	21.45 +/− 0.50	21.44 +/− 0.54

**Table 3:** Ablation study comparing single-task (STL), dual-task, and full multi-task (MTL) configurations. Bold values indicate the best result per metric. Verification margin is the mean negative L2 distance minus the mean positive L2 distance.

Configuration	Verification Acc. (%)	Classification Acc. (%)	F1 Macro	W- SSIM	PSNR (dB)	Verification Margin
V (Verification)	99.12	-	-	-	-	0.964
C (Classification)	-	99.38	0.994	-	-	-
R (Reconstruction)	-	-	-	0.482	24.29	-
V + C	99.17	**100**	**1**	-	-	0.894
V + R	99.17	-	-	0.482	24.32	0.999
C + R	-	99.07	0.991	0.48	24.29	-
V + C + R (Full MTL)	**99.81**	**100**	**1**	**0.485**	**24.36**	**1.028**

**Table 4:** KD loss-weight ablation: compactness, separability, and reconstruction quality.

Variant	Features Loss W	Logit Loss W	Temp.	Positive Distance	Margin	Norm Margin	Verif. Accuracy (%)	W-SSIM
No_KD	0	0	3	0.2276	0.9676	2.355	99.56	0.467
Feat_only	1	0	3	0.218	0.9682	2.486	99.61	0.471
Logit_only	0	0.5	3	0.2174	0.9761	2.451	99.71	0.469
Default	1	0.5	3	0.2116	0.98	2.454	99.46	0.468
Logit_heavy	0.3	1	3	0.2071	0.9858	2.474	99.81	0.469
Feat_heavy	2	0.15	3	0.2238	0.9703	2.414	99.66	0.462

**Table 5:** Reconstruction validity study: proposed model vs. unconcealed baseline across two evaluation modes using perception-related measures.

Metric	Proposed (Concealed Input)	Proposed (Unconcealed Input)	Autoencoder (Concealed Input)	Autoencoder (Unconcealed Input)
W-SSIM	0.468	0.428	0.216	0.991
PSNR (dB)	24.21	23.72	19.93	41.43
Edge SSIM	0.223	0.193	0.111	0.987
Edge MAE	0.086	0.090	0.100	0.011
Emb. Cosine Sim	0.965	0.940	0.928	1.000
Ver. Acc (%)	99.27	99.27	99.12	99.12
Cls. Acc (%)	99.38	99.38	99.38	99.38

**Table 6:** Anatomically based regional SSIM and PSNR breakdown under concealed input evaluation. Five facial regions are compared between the proposed model and the unconcealed baseline.

Region	Proposed SSIM	Autoencoder SSIM	Proposed PSNR (dB)	Autoencoder PSNR (dB)
Upper Left (eye)	0.537	0.27	24.35	19.94
Upper Right (eye)	0.515	0.265	24.04	19.82
Center (nose)	0.483	0.173	22.21	18.08
Lower Center (mouth)	0.449	0.185	23.45	19.28
Periphery	0.495	0.23	25.49	20.98

**Table 7:** Cross-attention fusion ablation study. W-SSIM = windowed SSIM (11 × 11 Gaussian kernel). Dist. Margin = mean negative L2 distance minus mean positive L2 distance.

Fusion Strategy	Description	Verif. Accuracy (%)	Class. Accuracy (%)	W-SSIM	PSNR (dB)	Dist. Margin
No Fusion	Concat + linear projection	99.32	99.38	0.467	24.22	0.970
Vis-Guided Only	Q = Vis, K/V = THz	99.56	99.38	0.466	24.13	0.970
THz-Guided Only	Q = THz, K/V = Vis	98.98	99.38	0.466	24.18	0.961
Dual Cross-Attention	Symmetric bidirectional	99.27	99.38	0.469	24.18	0.967

**Table 8:** Distillation integrity ablation. Alignment metrics are reported in two spaces: the projection space where Equation (12)’s L2 loss operates, and the embedding space where task heads consume representations. Ver. Acc and W-SSIM are student-only (THz-only inference, teacher absent). Bold is best per column, and † is worst per column (highlights the random teacher dissociation between projection and embedding spaces).

Variant	CKA (proj)	*ρ* (proj)	Cos (proj)	CKA (emb)	*ρ* (emb)	Ret@5 (%)	Verif. Accuracy (%)	W-SSIM
Proposed	0.839	0.634	**0.948**	0.511	0.477	30	98.68	0.464
No Distillation	0.558	0.343	−0.036	0.554	0.514	20	99.17	0.467
Random Teacher	**0.927**	**0.917**	0.844	0.467 ^†^	0.335 ^†^	10 ^†^	99.56	0.468
Permuted	0.480	0.330	0.922	0.503	0.466	30	99.42	0.469

## Data Availability

The complete implementation code and trained weights are provided at https://github.com/noamberg/thz-face-distil (accessed on 13 January 2026). The facial dataset is currently not publicly available.
